# “I See What You Mean”—A Case Study of the Interactional Foundation of Building a Working Alliance in Care Decisions Involving an Older Couple Living with Cognitive Decline

**DOI:** 10.3390/healthcare11152124

**Published:** 2023-07-25

**Authors:** Elin Nilsson, Anna Olaison

**Affiliations:** Division of Social Work, Department of Culture and Society, Linköping University, SE-601 74 Norrköping, Sweden; anna.olaison@liu.se

**Keywords:** dementia, families, needs assessment meetings, social work, the working alliance, conversation analysis, case study

## Abstract

Background: Social workers have a key role in needs assessment meetings with families dealing with dementia, providing information, support, and advocacy, while also assessing needs and making decisions about care services for several parties. These contacts are especially important during the introduction of home care services, where often the person has previously relied on informal support from relatives. The needs assessment process entails the involvement of all present parties, with the aim to reach a mutual agreement, a *working alliance*, regarding which services to apply for. Purpose: The aim of this case study is to explore how the participants, by means of different conversational practices, jointly create a working alliance between the different parties in one family. The study provides insights into the process of co-constructing a working alliance in the needs assessment process for elder care services. Methods: This article addresses the process by which social workers build a working alliance in a multi-party conversation with a family living with cognitive decline; a meeting that lasted 50 min. In this case study, we benefit from an inductive and detailed conversation analytic methodology. The theoretical framework of working alliances in institutional interaction has informed the analysis. Results: The findings illustrate how the social worker in this case study involves all parties in the decision regarding care services and explores the use of the conversational practices of *mitigations*, *positive framing*, *adding information,* and *positioning,* as a “we” achieve mutual agreement toward the end of several sequences. Conclusions: Drawing on the results of this case study, we argue that multi-party interaction involving relatives enables diversity in role-taking, where the professional, for instance, can pursue a more empathic role. Also, our results indicate that minimal agreement to a proposal is sufficient in a multi-party interaction involving clients with cognitive decline.

## 1. Introduction

Social workers have a key role in needs assessment meetings with families dealing with dementia, where their task is to provide information and support and to enable advocacy when assessing needs and making decisions about elder care services. In Sweden, elder care services in the form of home care are often the main care provider [1,2], alongside informal care by friends or family members [3]. In Swedish elder care, the needs assessment process is governed by the Social Services Act (SSA) [4], which aims to ensure a reasonable standard of living for recipients. However, the SSA does not provide detailed guidelines concerning the procedure to be followed regarding needs assessment, or how elder care services should be organized and distributed. Instead, this is steered by politicians at municipal level [5]. Prior to elder care services being offered to persons living at home, they must undertake a needs assessment process where an evaluation of the clients’ care needs is undertaken according to the recent changes in the SSA, chapter 5, paragraph 10 [6], the care manager. In Sweden, care manager is the professional title used for social workers who work on assessing needs for elder care services. Henceforth, we will use the term care manager in this article. It is also obliged to offer support to family members who care for a relative. During the needs assessment process, information is gathered from the older person, their family members, and representatives from other health and care agencies [7,8]. Here, the care manager must consider and assess the care needs of the client, while also endeavoring to ensure the person’s right to self-determination [8,9].

For those living with cognitive decline or dementia, applying for elder care services can be a complex process and may involve the views of several different parties regarding the need for and scope of services, or even a questioning of the need for services altogether. This may pose challenges to care managers responsible for leading assessment meetings, as they must balance the right of the person with dementia to autonomy and self-determination with the family members’ needs for relief and support in their informal care duties [10]. Thus, the care manager must be an advocate for all parties in a family and at the same time take into consideration a holistic view of the situation in order to find the best solution for the family. Previous research has shown that the views of people with moderate or severe dementia often get overridden with regard to care decisions [10,11]. However, research to date has not considered the position of care managers in the building of working alliances or shared agreements with families living with cognitive decline or early-stage dementia in the context of care decisions.

In this case study, we focus on institutional interaction when analyzing how one social worker in Sweden accomplishes a working alliance within a multi-party needs assessment meeting involving a family living with cognitive decline. The aim is to explore how the participants, by means of different conversational practices, jointly create a working alliance between the different parties in the family. This case study benefits from conversation analysis methodology [12] and the theoretical framework of the working alliance; see [13,14,15,16].

### 1.1. Research on Care Decisions Involving Dementia

In older couples where one partner has dementia, the situation around care in the home can often be complex [10,17]. Social and medical care situations involving either or both parties can be straining for their relationship [18]. It can be difficult for the partner without dementia to adopt the role of a carer and simultaneously uphold their role as a partner [10,19]. Similarly, it can be hard for the person with dementia to come to terms with the diagnosis, the experience of memory loss, and perhaps also feelings of being a burden to family members because of the disease [20,21]. Adult children are the main group who provide informal care for their older parents [22]. Research has highlighted that carers who take the role of the speaker in the assessment conversation get their version of the family’s needs heard, and this also often contributes to more services being granted [8]. On the other hand, research also shows that active participation by family members during assessment meetings may jeopardize the self-determination and voice of the person with dementia [23,24].

Research investigating different aspects of care decisions in elder care involving couples has defined a variety of strategies for involving the person with dementia; see [8]. As the disease progresses, these strategies often have a typical transition toward shared or substitute decision-making and dependence on the person’s past preferences [24,25,26]. Sinclair et al. [27] emphasize that transitions in decision-making processes might not be linear or follow a predictable pattern that is in line with the progression of the disease. In their study, they identified several decision-making approaches used by couples living with dementia (independent, joint, supported, and substituted). Different approaches were found to intertwine in daily life and were dependent on individual, relational, and external factors. Other studies found that persons with dementia, in their early stages, can be capable of articulating what is important to them regarding their needs and preferences about both the content and the delivery of elder care services [28,29]. Persons with dementia were also found to be less satisfied with the decision-making process about elder care services compared to their carers [30], and they were unable to influence care decisions left to them due to a feeling of loss of control over their everyday life [31]. St-Amant [32] also found that decisions about elder care services might have negative consequences for persons with dementia living at home and with family members, which can cause conflict and caregiver stress within the family. However, we have little insight into the actual process leading up to these decisions being made, specifically in the early stages of dementia or cognitive decline. Research to date has not considered the role of the care manager in navigating the wishes and needs for support and services expressed by the different parties involved, something that we address in this case study.

### 1.2. The Theoretical Framework of the Working Alliance

We consider the theoretical framework of the “*working alliance*” to be valuable in understanding how a social worker reaches an agreement in needs assessment meetings with a family living with cognitive decline. The working alliance, also called the helping alliance, describes the professional–client relationship, which, if based on mutual trust and understanding, has a positive effect on the outcome. It originates from psychotherapy literature and is the most researched institutional interaction in social work so far; see [33,34,35]. However, therapy is only one field of practice within social work. The working alliance focuses on how relationships are established in institutional interaction, something that is central to all social work interaction and underpins the relevance of further research within different fields of practice. Bordin [13] defined the working alliance as consisting of three components: (1) mutual agreement on the goals of the helping process; (2) mutual agreement on the tasks in the ongoing meeting, such as cognitive processes and behaviors of achieving those goals; and (3) the emotional bond founded on mutual trust and confidence. Together, these components define the strength and quality of the alliance. Flückinger et al. [36] further highlight that the alliance represents a proactive collaboration between clients and social workers across sessions and in moment-to-moment interactions.

There is a growing body of empirical studies on the working alliance in social work; see [37,38]. Studies indicate that the quality of the professional–client alliance predicts outcomes, regardless of the treatment types, for example in interventions with parents where there is a risk for abuse in child protection services [38,39]. A common feature of these studies is that they highlight the importance of the early establishment of goals that are agreed between the social worker and client in order for the work to be effective [33,40]. Strong engagement from the social worker can resolve ambivalence and enable clients to make positive changes and thereby enhance success [38]. Another vital part of the working alliance is that the social worker needs to demonstrate both personal qualities and technical skills in order to accomplish an alliance [41]. Consequently, these studies also highlight that social workers can be “human” during interactions and express personal feelings and thoughts, and this can sometimes make a difference in the building of an alliance [34]. The combination of professional skills with kindness, respect, and understanding makes clients feel safe and better able to manage their situation [16].

Koprowska [16] highlights that prior research on the working alliance in social work has had some challenges. First and foremost, alliance research has been mostly quantitative and has focused on the initial stages of forming an alliance, and less so in the middle or at the end of the process. Even though these studies have been able to prove the effect of value for alliances, they do not show how an alliance can lead to better outcomes [37,42]. Results from qualitative studies on the working alliance have often been based on participant–observer perspectives on the client–social worker interaction [43]. In this current case study, we intend to contribute to the knowledge gap regarding studies on the working alliance by focusing on an area that, to our knowledge, has not previously been addressed within the research on institutional interaction; namely, needs assessment meetings between social workers, older couples living with cognitive decline or dementia, and family members. In this case analysis, we will scrutinize the conversational practices used by participants in an interaction to jointly construct a working alliance, while also focusing on the different phases of building the alliance. Benefitting from conversation analysis, we analyze the construction of working alliances within naturally occurring data from one case drawn from a larger corpus, which gives a unique insight into how a working alliance is created and unfolds in social work practice.

## 2. Method

### 2.1. Data

The overall dataset from which the analyzed case is drawn consists of 18 audio or video-recorded needs assessment meetings between social workers and older couples, sometimes involving other family members or other professionals. In total, the dataset consists of 8.3 h of recordings from four municipalities in Sweden. Informed consent was obtained by the participants both verbally, by the care managers when recording the meetings, and in written form. The couples were sent information about the study and informed consent forms for them to sign and return by post to the researcher, indicating their consent for the researcher to analyze their recorded meeting. Four of the invited couples declined participation after the assessment meeting and their recordings were deleted and excluded from the dataset.

In order to gain analytical depth, the analysis for this case study is based on one audio recording of a home visit with wife Diana, husband Olle, their adult son Hannes, and a care manager. All names are fictive. The total recording is 50 min long, and the section included for analysis is approximately 7,5 min long, between 1.05 and 8.39. Diana was at the time of the interview under investigation for dementia as she had shown symptoms of cognitive decline. However, she has not yet received a diagnosis, and, therefore, we refer to her condition as cognitive decline in this article. It is also important to note that during the meeting, neither the care manager, the son, nor the husband make reference to Diana’s cognitive state. Diana, for her part, seeks support from her son on several occasions during the conversation by looking for reassurance that her statements are correct. However, we cannot draw the conclusion that her cognitive state is the reason for this action.

### 2.2. Analytical Procedure

The analysis departs from the methodological and theoretical framework of conversation analysis (CA), a data-driven inductive analysis of the participants’ own understanding of the conversation [12]. The interaction was transcribed according to the Jeffersonian transcription system [44], see Appendix A, including details such as prosody, pauses, and overlaps. In the analysis, we also benefit from previous research on working alliances in social work, i.e., [16,43]. Previous research on the topic of working alliances and institutional interaction in general often drew on dyads, namely a client and a professional; see [35]. The case in focus for the analysis was chosen based on its complexity regarding different opinions among the participants about care needs. The case with four participants also provides us with a whole different landscape of working alliances to disentangle analytically. The choice to conduct a case study rather than analyze several meetings was to achieve an analytical depth of the process of reaching a mutual agreement, something that has rarely been presented in research on institutional interaction. As the analytical method of conversation analysis [12] is so rich in details, one meeting was estimated as sufficient and within the space limit for the article. The analyzed case has a clear structure with different phases, where various conversational practices are adopted, and a working alliance is created over time. The extracts chosen for analysis are taken from a long sequence of interactional events where the participants discuss a proposal to the couple regarding a service that provides prepared meals for lunch. In the extracts, there are several occasions where diverging stances between spousal partners are presented in sequences of both active and passive resistance to services offered by the care manager, with whom the son is affiliated.

We describe and analyze the different interactional practices used to accomplish a working alliance in different phases, focusing specifically on the care manager and the adult son, who initiated the meeting. The analytical procedure involved a process of both authors listening to the recordings repeatedly and reading the transcripts. The extracts that were analysed were translated from Swedish to English and all personal information has been changed. The original Swedish version is presented in Appendix B. The whole sequence in which they discuss the topic of prepared lunches, which is the focus of the analysis, takes place one minute into the meeting and lasts for seven minutes.

Drawing on findings from similar research on institutional interaction, i.e., [10], we focused specifically on the participants’ conversational practices of *mitigations*, *positive framing*, *adding information*, and *positioning* as a “we” as part of working alliance formation. In the analysis of the case, we distinguish between what we refer to as “interactional alliances” and “working alliances”. Interactional alliances refer to more momentary positionings, where two or several participants take a stance as a unit in the ongoing interaction; for instance, referring to themselves as a “we”, see [45,46]. Interactional alliances can be key elements for the formation of an overall working alliance. Working alliances refer to situations where all parties express mutual agreement on goals and on the tasks for achieving those goals, and where there is an emotional bond involving trust; see [13,14,15,16]. The agreement here goes beyond the ongoing interaction. Before moving on to the analysis, two figures of working alliances are presented below as illustrations, which were taken from the larger dataset concerning multi-party interactions between care managers and couples. The excerpts have been transcribed and translated without conversation analysis notation to save space.

EX A: ANJ: Anja, wife, RIC: Richard, husband with care needs, CaM: care manager
**ANJ**: *No but it, it feels [ju] (The Swedish adverb ‘ju’ used for marking common ground, similar to the English ‘you know’. It can also be used for creating social affiliation, as well as expressing stance in negotiations, see* [47]*.) like we can speak truthfully with you, and what we, how things are and that. And then we have [ju] established a very nice connection with you, we think. That feels good.*
**CaM**: *Oh, that is nice to hear, that’s great. Cause I, I really hope [ju] that you find that, eh, cause I find that, I want [ju] for us to have that dialogue, that we can be open and honest with each other.*
**RIC**: *That is [ju] good. Very good.*

EX B: RON: Ronja, wife, LIN: Linus, husband with care needs, CaM: care manager, HoC: home care nurse, OcT: Occupational therapist
**CaM**: *No. But, but let’s summarise briefly and then it is [ju] basically that, Linus, the help you’ve had previously kind of continues right.*
**LIN**: *We continue as before.*
**CaM**: *Yes.*
**HoC**: *(Laughter)*
**CaM**: *But with the change that they come in the morning and evening, is that right?*
**RON**: *What do you say Linus?*
**LIN**: *Yeah, yeah, let’s continue like that, and if it improves then we cut back, if it gets worse we can increase.*
**CaM**: *Then you will let us know, yes*.

## 3. Results

This section provides an analysis of how a care manager conducts a needs assessment meeting with Diana, a woman with cognitive decline, her husband Olle, and their son Hannes, and how they jointly manage to reach a working alliance regarding the first topic of the meeting, which is prepared lunches for delivery. The process will be presented in chronological order, moving from initial pre-established alliances involving only some of the participants to a weak working alliance involving additional participants, which over time improves in strength as all participants are included.

### 3.1. Pre-Established Alliances

In this home visit, the interaction begins with some informal small talk before the care manager introduces the aim of the meeting one minute into the meeting (lines 1–4). She provides information regarding who has initiated the meeting, namely the son Hannes, as well as his suggestion to discuss his parents’ need for help of some kind.

After the care manager has introduced the aim of the meeting, Hannes accepts his position as the initiator and takes the role of the speaker (line 10). First, he informs all the participants that this is not only his own idea: he and his brothers have previously discussed the issue with the parents. In these brief lines, we can identify at least two types of expressions of interactional alliances, or positionings as a “we”; see [45,48]. The first regards the care manager’s use of “we” when introducing the aim of the meeting (line 1), indicating a shared position or a unit, possibly between herself and Hannes or even including Diana and Olle. Expressions of a second interactional alliance can be found in Hannes’ turn (lines 10–13) where he introduces a shared “we” with his non-present brothers, referring to their previous conversations with their parents. These two alliances together make a strong unit that gives weight to the aim of the meeting—discussing the parents’ need for help. The care manager and Hannes take turns and collaborate in expressing their shared position regarding the goal of the meeting, which is also a keystone of working alliance formation; see [13,14,15]. However, at this point, Diana and Olle are not actively included in any type of alliance; rather, they are positioned as “you” (collective) by both the care manager and Hannes (lines 3, 12, and 13). Expressions of several different interactional alliances on the same topic but at different times and involving different members can also, possibly, improve the strength of the ongoing project of forming working alliances.

**Figure 1:**
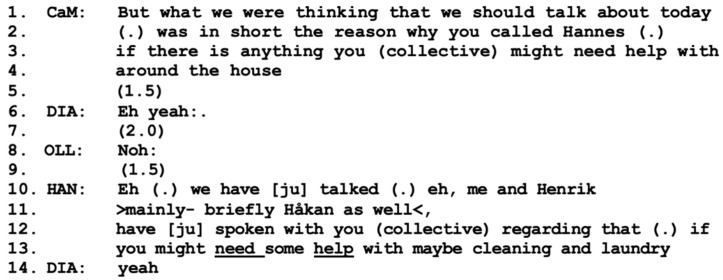
CaM (care manager), DIA (wife Diana), OLL (husband Olle), HAN (present son Hannes), Henrik and Håkan (non-present sons).

Hannes is given a central position by the care manager as she acknowledges that he initiated the meeting (lines 1–4). Hannes responds with a proactive stance as a spokesperson, see [36], departing from statements about his and his brothers’ previous actions and views rather than presenting the issue as coming from the parents, the “clients”. This is potentially a bold move by Hannes, as it may challenge the involvement of Diana and Olle, as well as their rights to first-hand knowledge regarding their own life and circumstances; see [49]. Hannes’ move can *challenge* the formation of a working alliance, as the parents are presented with a pre-established opinion or agenda in which they are treated as recipients (passive) rather than agents (active). However, this move can also have the beneficial consequence of *contributing* to the forming of a working alliance in which all are included, as the couple might be influenced by and depending on the shared view of all their sons. Hannes develops the content of the envisaged help by suggesting that his parents might need help “maybe with cleaning and laundry” (lines 12–13). This turn receives a brief “yes” from his mother, Diana, possibly indicating that she is familiar with the previous conversation. Taken together, several different interactional alliances and pre-established alliances can be seen in Figure 1. However, at this point, the clients, Diana, and Olle, are not included, resulting in a weak working alliance overall.

### 3.2. Diana Joins the Working Alliance and Improves Its Strength

**Figure 2:**
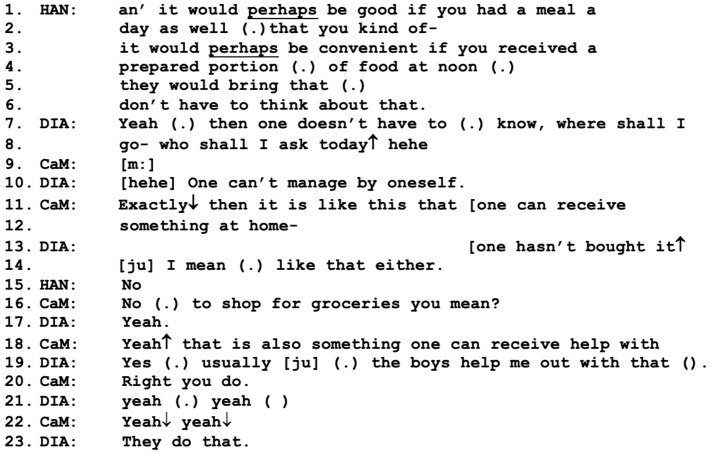
CaM (care manager), DIA (wife Diana), HAN (present son Hannes).

Figure 2 follows immediately after Figure 1. In this part, the strength of the working alliance increases as Diana expresses some affiliation and collaboration regarding the proposed service. Hannes introduces the main topic of prepared lunches with the positively framed proposal that it would “perhaps” (SWE: “kanske”) be “convenient” for them to receive prepared meals, so that they “don’t have to think about that” (lines 1–6).

Here, we can see conversational practices such as downplays, or *mitigations*, of the proposal, such as “perhaps” (lines 1, 3) as well as *positive framing,* such as “convenient for you” and “don’t have to think about that” (lines 3–6). Both types of conversational practices have been identified previously in the context of care managers and relatives introducing offers for elder care services to an older client, where they are often used to deal with actual or anticipated resistance [10]. In this figure, this mitigated approach by Hannes receives agreement from Diana, who approves of the idea of not having to think about who to ask for help every day, and in fact says that the couple cannot manage to prepare lunch themselves (lines 7–8, 10). Again, the care manager takes the role of the speaker by agreeing and then *adding information* from her professional perspective, informing them about the service that they are eligible to apply for (lines 11–12, 18). Here, the care manager balances the act of providing information about the available service with listening to and not questioning or correcting Diana when she describes the current situation of receiving informal help with shopping (lines 15–19). Diana’s approach may be seen as a demonstration of what Horvath and Greenberg [15] describe as key elements of forming a working alliance; namely, supporting a good relationship with the client as well as making use of professional skills. Also, it may contribute to what Bordin [13] and Koprowska [16] refer to as an emotional bond based on kindness, respect, and understanding, which is also important for working alliance formation. Taken together, the three active participants in Figure 2—Hannes, the care manager, and Diana—reach a mutual agreement, a working alliance, regarding the benefits of receiving prepared lunches, whereas Olle stays silent during this exchange.

### 3.3. Olle Resists the Proposal

**Figure 3:**
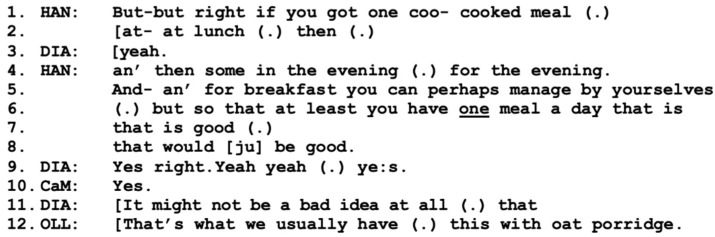
CaM (care manager), DIA (wife Diana), OLL (husband Olle), HAN (present son Hannes).

Figure 3 follows immediately after Figure 2. Here, Diana and the care manager continue their conversation about how the “boys” (the sons) help the parents with shopping (not included here) before Hannes reorients the participants to the topic of his parents receiving prepared lunches (line 1 below).

The way Hannes returns to the topic of prepared meals clearly indicates that from his perspective, this is the main issue for the meeting, rather than additional small talk. He also makes visible that he believes that his parents are capable of preparing both breakfast and evening meals by themselves, but again emphasizes the benefits of receiving a proper meal at lunchtime (lines 6–8). Diana clearly affiliates by stating that it “might” be a good idea (lines 9, 11), whereas Olle, in overlap, instead focuses on what they normally eat (for breakfast supposedly), porridge (line 12). In this figure, Hannes takes an active position in pursuing the proposal of prepared meals, whereas the care manager takes a rather passive position, which is possibly due to his already expressed agreement with the proposal.

**Figure 4:**
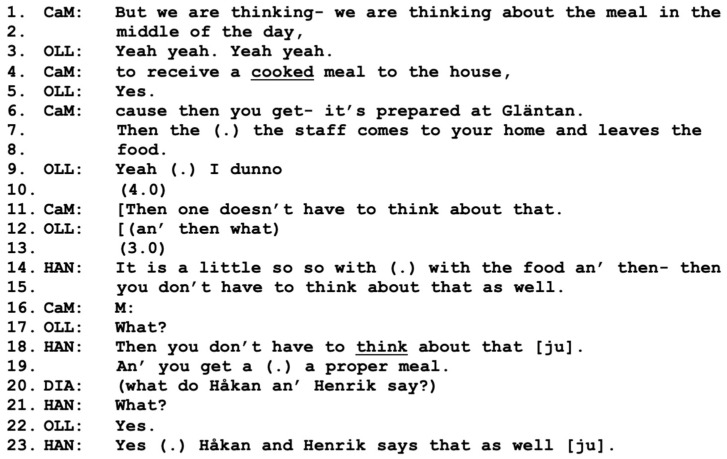
CaM (care manager), DIA (wife Diana), OLL (husband Olle), HAN (present son Hannes), Henrik and Håkan (non-present sons).

In the first three figures, we see that three out of four participants have reached a mutual agreement, a working alliance regarding the benefits of prepared lunches, as well as agreement about the underlying need for them. However, the working alliance still excludes Olle, who has not yet participated at all in the shared agreement. This puts the care manager in a difficult position, as she needs both Olle and Diana to be on board with the working alliance in order for her to continue with an application for social services [9]. This is possibly why she returns to the topic of prepared lunches in response to Olle’s comment about porridge; see line 1 in Figure 4 below.

When returning to the topic of prepared lunches, the care manager positions herself within an interactional alliance of “we” who are thinking about the midday meal, rather than as a single actor (line 1). It is not clear at this point who is considered as part of the interactional alliance “we”, but the care manager’s turn here has a similar design as in Figure 1 when she started the meeting. However, in Figure 4, Diana may be included in the “we” as she has explicitly expressed a positive assessment of the proposal. This turn by the care manager (line 1) is the continuation of a repair sequence in response to a misunderstanding about which meals they are discussing, where Olle had continued to talk about porridge, and it had been clarified that they were discussing lunch (not included). This may be the reason why Olle responds “Yeah yeah. Yeah yeah” (line 3). The care manager potentially treats Olle’s turn as an agreement to the service and even potentially an alliance, as she moves on to describe how the service works (lines 6–8). Here, Olle for the first time expresses some resistance to the proposal with his “yeah (.) I dunno” (line 9); see [10] on resistance). This resistance is not taken up by the other participants; instead, there is a four-second silence. After this silence, the care manager, yet again, *describes positive aspects* of the service (line 11), possibly a conversational practice for meeting the resistance shown by Olle [10]. Her turn happens to overlap with a potentially challenging turn by Olle, “an’ then what” (line 12). Again, the resistance by Olle is not explicitly responded to or topicalized; instead, there is another long silence before Hannes responds, motivating the service by reusing the positively charged statement made by the care manager, “then you don’t have to think about that” (lines 14–15, 18), which clearly positions him in agreement and as a collaborator in alliance with the care manager.

**Figure 5:**
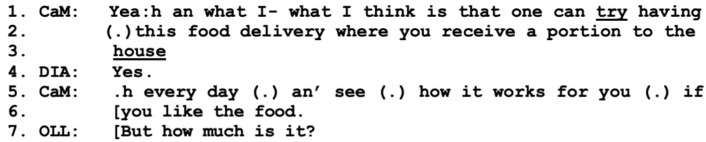
CaM (care manager), DIA (wife Diana), OLL (husband Olle).

Interestingly, Diana takes the role of the speaker here by asking Hannes what his brothers think about the proposed service (line 20). We cannot know why she asks Hannes this question at this point: whether she is not sure how she feels about the proposal herself and needs more input from other perspectives, or whether she is attempting to build a stronger case involving all the sons in order to get her husband on board. Hannes affirms that both his brothers agree with him that the service is a good idea (line 23); however, he does not specify the details of what his brothers think regarding the topic. Hannes continues to mention additional benefits, such as access to nutritious food (excluded from the transcript), before the care manager takes the role of the speaker again in line 1 in Figure 5 below.

The care manager returns to the topic of prepared lunches and makes a clearly stated proposal, now from the position of “I” rather than “we” (line 1). Here, she clearly takes the position of the one in charge; however, as her turn follows Hannes’ comments about nutrition (excluded), we can see that it is a co-construction, as she agrees with Hannes “Yea:h” (line 1) and adds information from her perspective as a professional. She describes the terms for the service (lines 1–3), as well as provides information about the couple’s possibilities for self-determination: “an’ see how it works for you (.) if you like the food” (lines 5–6). Thus, the responsibilities for pursuing the topic of prepared meals have shifted somewhat from Hannes to the care manager, while the working alliance remains clear and strong in their collaborative pursuit of the proposal.

**Figure 6:**
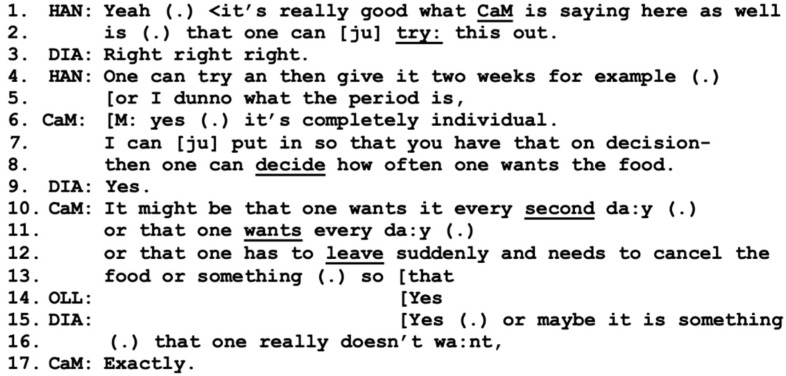
CaM (care manager), DIA (wife Diana), OLL (husband Olle), HAN (present son Hannes).

When the care manager describes the possibilities open to the couple, she speaks in overlap with Olle, who poses a question about the costs for the service (line 7), clearly indicating a hesitant stance, as he starts with a “but”. This indicates that the parties have not yet reached a working alliance in which all are included. The answer to Olle’s question is provided by the care manager (not included), and Hannes adds to the conversation by raising the issue of costs for groceries if they cook themselves before he returns to the issue of the meal service; see below in Figure 6.

### 3.4. Managing Olle’s Resistance

At this point, the participants have been discussing the issue of prepared lunches for some minutes, but before moving on to other issues, the care manager and Hannes need to get all participants on board with the proposal. Hence, there is a need to manage the resistance shown by Olle and get him included in the working alliance.

Hannes makes a positive assessment regarding the information given by the care manager about the opportunity to try the service out (lines 1–2). Hannes receives agreement from Diana (line 3), which may encourage Hannes to continue and develop a more detailed proposal of a two-week trial period; however, he makes an epistemic disclaimer, stating that he is not an expert on the topic [49]. Instantly, the care manager adds the required information from her perspective as a professional (lines 6–8, 10–13), which neatly interweaves with the information provided by Hannes. During these lines, the collaborative approach between Hannes and the care manager indicates mutual trust and confidence that they are “on the same page” [15], which has now been built up over several interactional sequences [36]. It is almost as if Hannes takes on the role of co-care manager, indicating a strong working alliance between these two participants where this trespassing of responsibilities is allowed.

**Figure 7:**
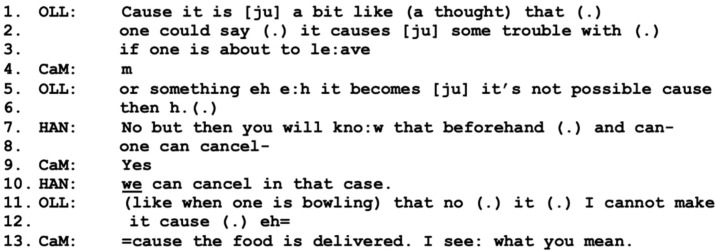
CaM (care manager), OLL (husband Olle), HAN (present son Hannes).

The information given about the service, however, receives minimal agreement by Olle (line 14), while Diana, in overlap, adds information about the possibilities of the service (lines 15–16). This turn is not taken up; instead, Olle takes the role of the speaker by describing a hypothetical problematic situation where they would not be home at lunchtime (lines 1–3, 5–6), below in Figure 7.

**Figure 8:**
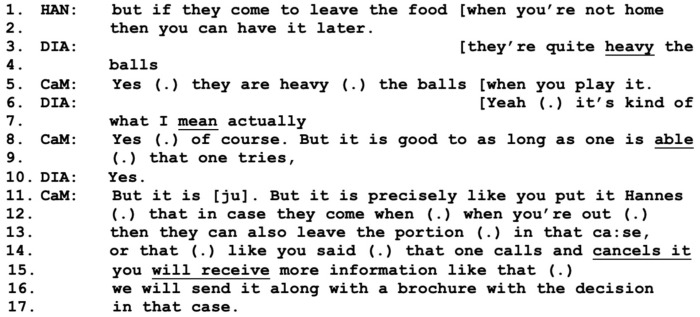
CaM (care manager), DIA (wife Diana), HAN (present son Hannes).

In response to Olle’s hypothetical problem, Hannes instantly downplays the issue by stating the possibility for “one” (SWE: “man”) to cancel the delivery (lines 7–8), a turn that receives agreement by the care manager. Hannes then reformulates by emphasizing that “we” can cancel it (line 10), which may be a conversational practice of mitigating the effort of cancellation for Olle, suggesting that Hannes himself can share responsibility for the task. Olle then further refers to a hypothetical situation where he might be out bowling at the time of delivery (lines 11–12). The care manager responds by adding to and finishing Olle’s turn, while also expressing understanding of Olle’s concerns: “I see what you mean” (line 13). This empathic approach can be one important part of including Olle in the working alliance and thereby also increasing the strength of it; see [13]. This sequence is followed by a turn by Diana, where she jokingly proposes that Olle might be too old for bowling anyway, to which Olle responds in a similarly humorous tone (not included). This small talk is again cut off by Hannes, who returns to addressing the issue of Olle not being home for lunch by suggesting that he can simply have the food when he gets back (Figure 8-line 1).

In the meantime, Diana continues to overlap on the topic of bowling in a side sequence with the care manager (lines 3–10), where the care manager expresses positivity and understanding regarding Olle’s intentions to stay active. She then returns to Hannes’ comments about the prepared lunches (lines 11–17) by verifying that they can indeed be left for the couple even if they are not home. However, she also returns to the issue of cancellation, which both Olle and Diana have raised previously, reassuring them that this is also an option, and that further information will be sent to them in case of a decision. In this sequence, the care manager uses both professional and personal skills and shows respect and understanding, see [16,36,41], as she successfully wraps up the sequence by responding to the views and concerns expressed by Hannes, Diana, and Olle. This act of balance by the professional, the care manager in this case, may influence or even predict the outcome of the meeting, see [37], which, in the long run, may also contribute to change in the situation for all parties [38] as well as benefit a strong working alliance [16].

### 3.5. Agreement Based on a Weak Working Alliance

When wrapping up the discussion about prepared lunches, the participants finally accomplish a working alliance that includes Olle, albeit of a weak kind.

**Figure 9:**
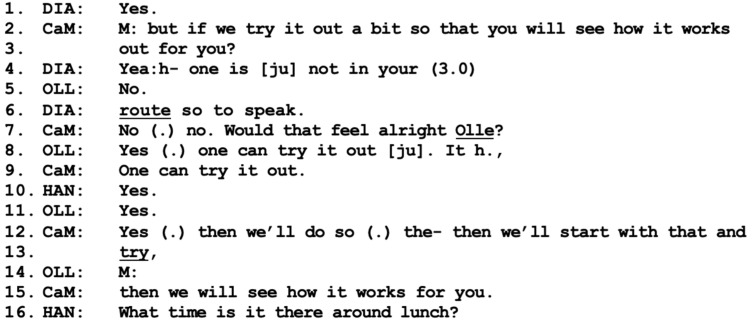
CaM (care manager), DIA (wife Diana), OLL (husband Olle), HAN (present son Hannes).

As the participants reach the end of the discussion on meal deliveries, the care manager again uses the communication practice of a mitigated formulation, “try it out a bit” (SWE: “testar lite”), in her question addressed to Olle and Diana as a collective “you” (Figure 9-lines 2–3), a practice that is commonly used when there is some type of resistance [10]. Diana instantly expresses a willingness to accept the proposal, whereas Olle provides a response that is more difficult to interpret (line 5). This is probably why the care manager specifically addresses Olle on line 7, asking “would that feel alright Olle?”, and Olle now indicates agreement to try it out (line 8). Here, it is clear who oversees pursuing and wrapping up the institutional agenda, namely the care manager. Hannes takes a rather passive position in this final sequence and speaks only to ask for details regarding the service (line 16). At this point, all parties have reached a mutual agreement on both the goals of the helping process and the delivery of the service and have all been heard and acknowledged in different phases of the conversation. Drawing on these cornerstones, we can assume that they have accomplished a working alliance that includes all participants; see [13]. However, the strength of the alliance is still rather weak, as Olle has not contributed with collaborative turns or explicitly expressed positive stances toward the proposed service.

## 4. Discussion

In this case study, we demonstrate the co-construction of a working alliance involving all parties of a needs assessment meeting. The agreement, or working alliance, is accomplished over time by the use of the conversational practices of *mitigations*, *positive framing*, *adding information,* and *positioning* as a “we”. In institutional encounters, the professional, in this case the care manager, has a key role in pursuing the agenda of the meeting and accomplishing a shared agreement regarding services. However, in this case study, we present figures in which the adult son, Hannes, takes over some of the responsibilities for presenting and pursuing the agenda, which enables space for the care manager to adopt a more relational and empathic approach to the couple; see [16,36,41]. Throughout the meeting, the care manager attends to the communicative contributions of both the older adults, Diana and Olle, validating their views without challenging the course of the meeting. Hannes, on the other hand, has a clear focus on talking about his parents’ potential benefits from receiving elder care services, clearly in alliance with the comments of the care manager. Looking at the participants’ role-taking [50] and status [51], Hannes may also be seen as a relative in need of some relief from caring for his parents. However, in the context where his parents are present, his alliance with the social worker, both pre-established and presently co-constructed, provides him with a more deontic status, see [51], which is more in line with the professional status of the care manager.

Previous research has shown that social workers often must coax and lure clients who express resistance to services that are deemed beneficial or necessary for them and/or their relatives [52]. In a similar way as shown here, longer sequences of persuasion or forming of working alliances may result in only minimal agreement from the older adult; see [10]. This puts social workers in a situation of having to continue to ask for agreement or acceptance in order for them to pursue an application for a service, as in Sweden they require a clearly stated wish for this by the client with care needs [53]. In this case study, the end of the discussion on prepared meals resulted in a weak alliance, which nonetheless was apparently deemed good enough by the social worker as she went ahead with the application for the service. This result is in line with previous research on shared decision-making, which shows that the views of persons with dementia can be heard, but that the outcome also depends on social factors and the contributions of other participants; see [27]. The debate on shared decision-making in adult social work has highlighted the potential of the frameworks (supported, shared, and substituted decision-making) in varying ways to support an individual in making decisions. However, research to date has mainly revolved around the development and principal ideas of the conceptual frameworks themselves and how they can be applied in practice [54,55]. Nevertheless, there is still a lack of empirical evidence regarding how these frameworks are implemented and developed in practice. As such, the results of this case study and other current studies, see [10,56], highlight the complexity of ensuring self-determination for people with dementia in multi-party interactions. The results specifically point to the relation between working alliances and supported decision-making in practice, a finding that adds to the current debate in social work.

The stage of cognitive decline or dementia may also be worth considering in research on working alliances within institutional interaction. In the present case study, the dialogue is dominated by talk between Diana, Hannes, and the social worker, whereas Olle has a more passive role, only occasionally raising questions or expressing hesitation in regard to the service in question. Diana is under assessment for dementia, which indicates an early stage of a potential cognitive decline or dementia. Research involving persons with severe dementia shows that the involvement, epistemic primacy, and status of the person with dementia are heavily challenged during needs assessments; see [8,10]. However, here, Diana’s potential cognitive decline is not referred to by the other participants, nor is it evident in her communicative contributions. Instead, it is the resistance expressed by her partner, Olle, which poses the greatest challenge to the formation of a working alliance. Diana expresses a level of trust in the views expressed by the care manager and her son and even requests information about the views of her other sons. The reason for this cannot be stated with certainty, but an incipient cognitive decline can lead a person to trust close relatives more than themselves and potentially hand over speakership and important decisions to them; see [57]. On the other hand, relational factors and connecting with both the social worker and the views of relatives have been shown to be specifically important for women living with cognitive decline or dementia; see [58]. Our study contributes to the research on institutional interaction involving older couples with dementia [10,11], as it demonstrates the building of a working alliance in a needs assessment meeting involving a woman with potential cognitive decline in an early stage. We have shown that social workers can enhance self-determination for a person with cognitive decline by using and combining interactional, professional, and technical skills with personal, relational, and empathic qualities, which are all deemed important elements in building a working alliance [16].

The findings from this case study can possibly also make a contribution to the field of institutional interaction and working alliance formation, which highlights the importance of strong engagement from the social worker to enable clients to make positive changes [38]. However, most research on this topic draws on dyadic interaction, see [35,43], involving a client and a professional, and has been carried out in relation to other helping relationships than assessment meetings. In social work and other people-oriented practices, meetings often involve several other parties. For instance, needs assessment meetings often involve other professionals, such as a nurse or an occupational therapist, or a social worker from another area, who all have different aims for the meeting, experiences, and areas of expertise. Also, present or non-present family members may have different views and aims, as well as several different needs for care that must be considered. In assessment meetings, there is a possibility, or sometimes an obligation, for the professional to take sides (implicitly or explicitly) with one of the parties when discussing and making decisions that are considered most suitable for all. Looking at multi-party interaction from this perspective, we argue that applying the theoretical framework of the working alliance is fruitful, as it can reveal how social workers navigate the task of building an alliance with both parties in an older couple as well as with participating relatives. This is a complex practice involving social workers’ professional skills, respect and understanding for both the couple living with cognitive decline and the whole family’s situation, and the ability to follow legislation and rules to be able to make an assessment. From this perspective, this paper adds to existing theoretical knowledge about the working alliance as it applies it to multi-party interactions and contributes new knowledge about the shared interactional accomplishments of the participants.

Severe dementia may also pose challenges in terms of a person’s ability to remember the agreements that have been made, as well as for other participants to anticipate and take for granted the person with dementia’s current knowledge regarding the agreements; see [57]. Further research on multi-party interactions involving people living with dementia at different stages is needed, specifically in institutional interactions involving major life changes, either medical or social. It would be beneficial to conduct research on a larger dataset, as this could contribute to greater diversity in the methods of alliance formation. Another focus for future research could be to involve other groups of persons with cognitive decline where, for example, progression to severe dementia may drastically change the participation framework, as well as the severity of the decisions for the person with dementia.

## 5. Conclusions

Benefitting from detailed conversation analysis, this case study shows that the accomplishment of reaching a mutual agreement, a working alliance, can depend on several interactional aspects and commitment by all present participants. In relation to institutional interaction, such as needs assessment meetings, a multi-party interaction that involves several family members rather than just the client may enable the professional to navigate more freely and take different roles throughout the meeting. As shown here, a more empathic and inclusive approach from the professional can be beneficial for reaching a mutual agreement and the provision of adapted care and appropriate elder care services.

## Figures and Tables

**Figure A1:**
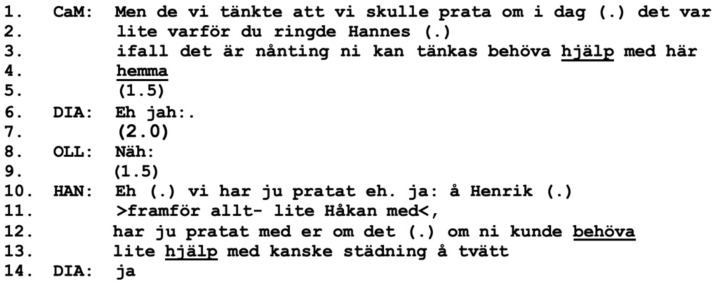
CaM (biståndshandläggare), DIA (hustru Diana), OLL (man Olle), HAN (närvarande son Hannes), Henrik and Håkan (ej närvarande söner).

**Figure A2:**
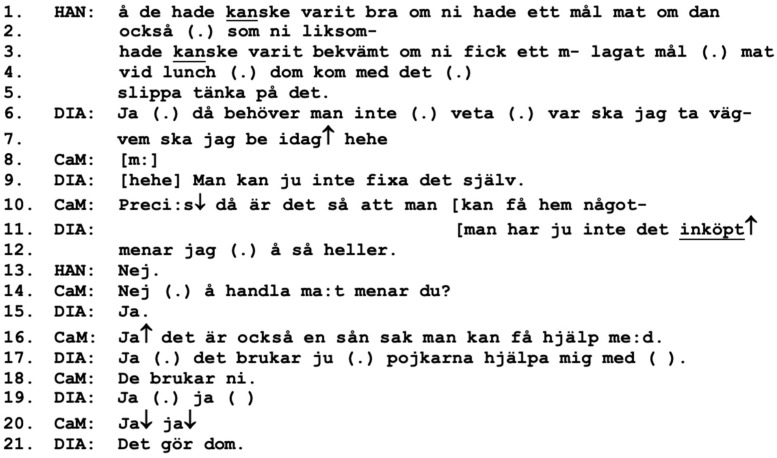
CaM (biståndshandläggare), DIA (hustru Diana), HAN (närvarande son Hannes).

**Figure A3:**
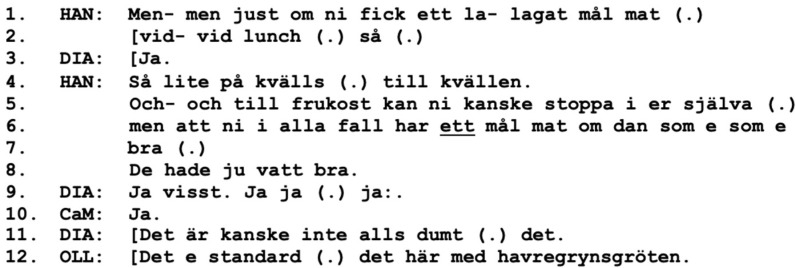
CaM (biståndshandläggare), DIA (hustru Diana), OLL (man Olle), HAN (närvarande son Hannes).

**Figure A4:**
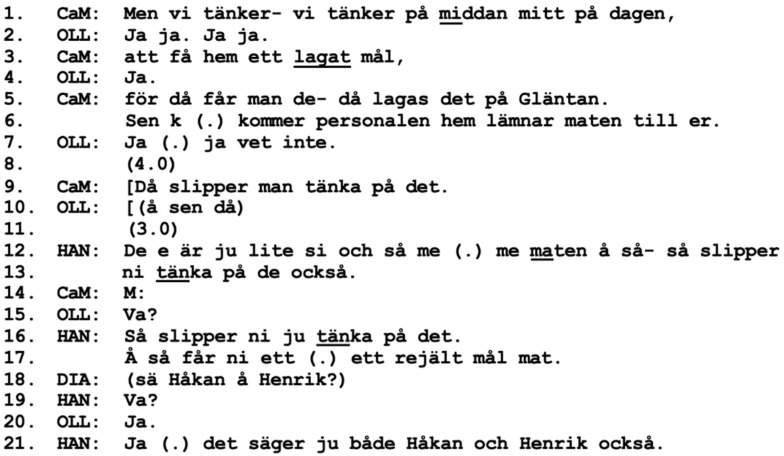
CaM (biståndshandläggare), DIA (hustru Diana), OLL (man Olle), HAN (närvarande son Hannes), Henrik and Håkan (ej närvarande söner).

**Figure A5:**
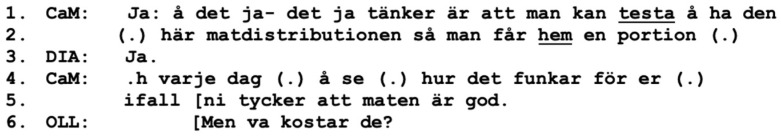
CaM (biståndshandläggare), DIA (hustru Diana), OLL (man Olle).

**Figure A6:**
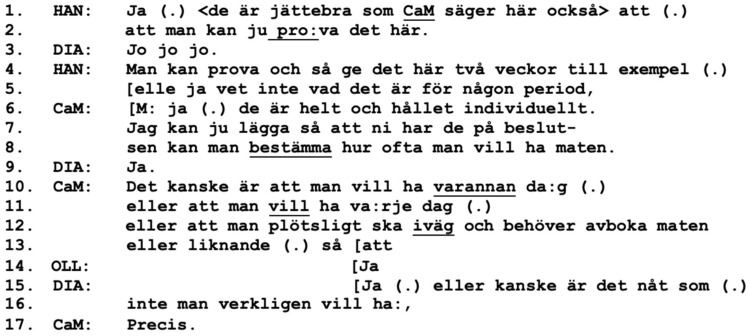
CaM (biståndshandläggare), DIA (hustru Diana), OLL (man Olle), HAN (närvarande son Hannes).

**Figure A7:**
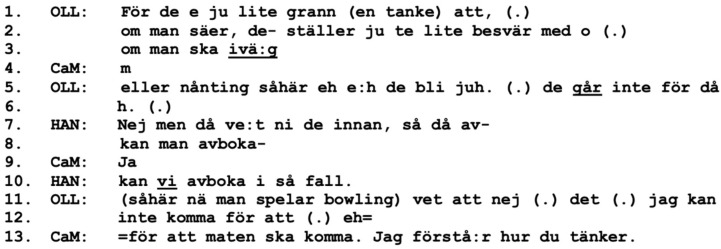
CaM (biståndshandläggare), OLL (man Olle), HAN (närvarande son Hannes).

**Figure A8:**
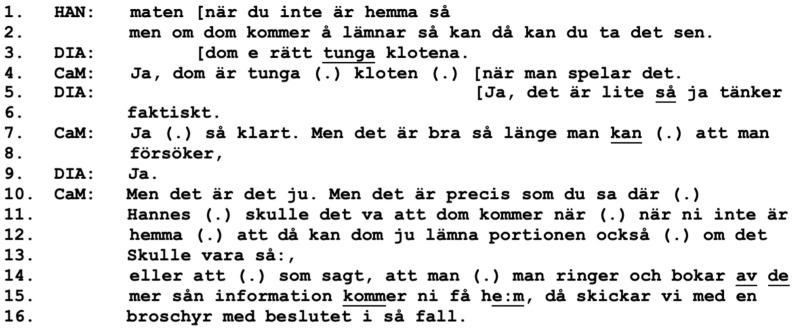
CaM (biståndshandläggare), DIA (hustru Diana), HAN (närvarande son Hannes).

**Figure A9:**
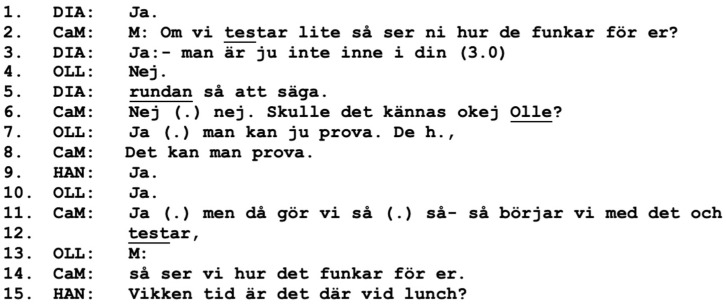
CaM (biståndshandläggare), DIA (hustru Diana), OLL (man Olle), HAN (närvarande son Hannes).

## Data Availability

The data are not publicly available due to privacy and ethical restrictions.
